# Hepcidin and Iron Metabolism in Pregnancy: Correlation with Smoking and Birth Weight and Length

**DOI:** 10.1007/s12011-016-0621-7

**Published:** 2016-01-20

**Authors:** Magdalena Chełchowska, Jadwiga Ambroszkiewicz, Joanna Gajewska, Ewa Jabłońska-Głąb, Tomasz M. Maciejewski, Mariusz Ołtarzewski

**Affiliations:** 1 Screening Department, Institute of Mother and Child, Kasprzaka 17a, 01-211 Warsaw, Poland; 2 Department of Obstetrics and Gynecology, Institute of Mother and Child, Kasprzaka 17a, 01-211 Warsaw, Poland

**Keywords:** Hepcidin, Tobacco smoking, Pregnancy, Birth body weight, Birth body length

## Abstract

To estimate the effect of tobacco smoking on iron homeostasis and the possible association between hepcidin and the neonatal birth weight and length, concentrations of serum hepcidin and selected iron markers were measured in 81 healthy pregnant women (41 smokers and 40 nonsmokers). The smoking mothers had significantly lower concentrations of serum hepcidin (*p* < 0.001), iron (*p* < 0.001), and hemoglobin (*p* < 0.05), but higher erythropoietin (*p* < 0.05) levels compared with non-smoking pregnant women. Logistic regression analysis showed the highest negative impact of the number of cigarettes smoked per day (*β* = −0.46; *p* < 0.01) and positive impact of ferritin level (*β* = 0.47; *p* < 0.001) on serum hepcidin concentration. The birth weight and the body length of smoking mothers’ infants were significantly lower than in tobacco abstinent group (*p* < 0.001). In multiple regression analysis, birth body weight (*β* = 0.56; *p* < 0.001) and length (*β* = 0.50; *p* < 0.001) were significantly related to maternal hepcidin values. Tobacco smoking affected hepcidin level in serum of pregnant women in a dose-dependent manner. Low concentrations of iron and hemoglobin in maternal serum coexisting with high level of erythropoietin suggest that smoking could lead to subclinical iron deficiency and chronic hypoxia not only in mothers but also in fetus. Low serum hepcidin concentration in smoking pregnant women might be associated with lower fetal birth weight and length.

## Introduction

Iron deficiency during pregnancy continues to be a common clinical problem and is one of the most prevalent nutritional deficits both in the industrial and developing countries. Iron requirements increase significantly (near 10-fold) over gestation; therefore, pregnant women are particularly at risk of developing iron deficiency anemia. The prevalence of anemia is dependent on nutritional status and use of sufficient prenatal supplements and ranges from 14 to 52 % women who are not taking prenatal supplements, to 0–25 % among pregnant women receiving regular multivitamin (containing iron and folic acid) preparation [1–5].

In the last decade, much attention was paid to research on new markers involved in the regulation of iron homeostasis [4, 6]. Hepcidin, a 25-amino-acids peptide hormone (Hep-25), has recently emerged as a key regulator of iron homeostasis. Apart from main synthesis in the liver, hepcidin messenger RNA (mRNA) is also expressed in the lung, heart, brain, and placenta tissue. Hepcidin regulates intestinal absorption, macrophage release, and the placental passage of iron. This hormone inhibits the cellular efflux of iron by binding to and inducing the degradation of ferroportin, the only known cellular iron exporter [7]. The role of hepcidin in the regulation of iron metabolism in pregnancy and its effects on the fetus and consequently on the newborn is not fully understood. Rising iron requirements in subsequent trimesters of pregnancy, in both the mother and the fetus, induces an increase in maternal dietary iron absorption and increase iron flux to the fetus via placenta [1, 2]. In utero-placenta unit, hepcidin causing degradation of syncytiotrophoblast ferroportin regulates iron release into the fetal circulation [8, 9]. Hepcidin expression is induced by iron overload and inflammation and is suppressed by anemia, erythropoietic activity, and hypoxia [10, 11]. Iron deficiency in pregnant women is assumed to be enhanced by cigarette smoking [12, 13]. Despite the fact that in the last years, a decrease in the number of active smokers has been observed and knowledge of the adverse health effect of smoking during pregnancy is quite common, 25–30 % pregnant women in Poland continue smoking [14, 15]. Tobacco smoking is associated in dose-related manner with stimulation of erythropoiesis due to increased maternal and fetal iron requirements probably through a hypoxic effect [16–18]. It is suggested that accompanying smoking hypoxia can suppress hepcidin mRNA expression and reduces hepcidin concentration in pregnant women as well as in newborns.

The aim of the study was to estimate the effect of tobacco smoking on serum hepcidin and selected iron parameters in pregnant women. We evaluated relationships between hepcidin and other indicators of iron status. Furthermore, we investigated the possible association between serum hepcidin concentration and the neonatal birth weight and length.

## Methods

### Patients

Two groups of women were taken from a prospective observational study for adverse effects of tobacco smoking in pregnant women visiting the Department of Obstetrics and Gynecology, Institute of Mother and Child, Warsaw, Poland, between January 2013 and March 2015. The study was conducted according to the principles of the Declaration of Helsinki and was approved by the Ethics Committee of the Institute of Mother and Child. All pregnant volunteers were made aware of the study objectives and informed written consent was obtained for analysis of biological samples and linking results to the data collected from questionnaires.

The base study population, wherein the present study was nested, constituted 296 singleton pregnancies. In the study group, 41 cases were included which had hospital visits in the third trimester of pregnancy (32–36 weeks) and continued smoking during pregnancy (minimum five cigarettes per day and minimum 2 years before conception). The control group consisted of 40 non-smoking, healthy pregnant women similar to age and gestational age. Gestational age was estimated by the last menstrual period and confirmed or corrected by ultrasonographic measurements of the crown-rump length (dated in the first trimester). The groups included only women with a single physiological pregnancy who, before pregnancy, had normal menstrual cycles, were in good health at time of examination, and declared good living conditions. The exclusion criteria for the study were maternal diseases (preeclampsia, hypertension, diabetes mellitus, active hepatitis, renal and cardiovascular diseases, and inflammatory conditions), multiple pregnancy, birth defects detected during pregnancy, alcohol drinking, and assisted reproduction. Women exposed to second-hand smoke (smoking spouse or other family members, co-workers) were excluded from the tobacco abstinent group. The classification of groups was confirmed by measurement of serum cotinine- the major metabolite of nicotine in pregnant women. A cutoff value of ≥13.7 μg/L was used to separate smokers from nonsmokers, in accordance with others studies [19]. All subjects in our study reported taking standard vitamins with iron and folate during pregnancy (average 400–800 μg of folate, 15–60 mg of ferrous fumarate daily).

After birth, clinical information on the infants, including weight and body length, were collected.

### Collection and Analysis of Blood Samples

Maternal peripheral blood samples (5 mL) were taken at the time of recruitment (30–34 weeks of gestation). In order to obtain serum, the blood was centrifuge at 2500×*g* at 4 °C for 10 min and was stored in small portions at −25 °C for subsequent biochemical analysis.

Serum hepcidin, erythropoietin (EPO), and soluble transferrin receptor (sTfR) values were determined by immunoassay (DRG, Marburg Mediagnost, Reutlingen, Germany). The limit of detection was 0.35 ng/mL for hepcidin, 1.1 mIU/mL for EPO, and 2 ng/mL for sTfR, respectively. The intra- and inter-assay coefficients of variation were less than 3.3 and 11.5 % for hepcidin, 8.4 and 8.8 % for EPO, and 6.0 and 7.0 % for sTfR, respectively.

Serum levels of iron (Fe), ferritin (Ft), transferrin (Tf), and C-reactive protein (CRP) were measured by standard methods using commercially available kits on COBAS INTEGRA analyzer (Roche, Switzerland). Concentration of hemoglobin (Hgb) in blood was determined using commercially available kits on Pentra 60 hematology analyzer (HORRIBA ABX, France).

Cotinine levels in serum were evaluated by immunoenzymatic method using a commercially available kit Cotinine one-step ELISA (Calbiotech Inc., Spring Valley, CA, USA).

Pre-pregnancy body mass index (BMI) was calculated using height and pre-pregnancy weight. Newborn infants were examined in the first 24 h of life. Neonatal length and weight were determined using a measuring board to the nearest 0.1 cm and a calibrated scale to the nearest 10 g.

### Statistical Analysis

Statistical analysis was performed using STATISTICA 10.0 (StatSoft, Poland) software. Data were presented as mean values and standard deviation (SD) for normal distribution or median value and interquartile range (25th–75th percentiles) for skewed distribution. The Shapiro-Wilk test was used for evaluation normality of data distribution prior to statistical analysis. Group differences were determined by Student’s *t* test or Mann-Whitney test depending on the assumptions. The chi-square test was used for comparing nominal variables. For simple correlation analysis, Pearson or Spearman correlation coefficients were calculated. Linear regression models with concentration of hepcidin as a dependent variable were estimated in the separate sample of smokers to examine the potential impact of some iron parameters, number of the cigarettes per day, and cotinine level. To evaluate relationships between anthropometric parameters and studied iron markers, multivariate linear regression models with body birth weight and body birth length as dependent variables were estimated (adjusted for gender and smoking status).

Results were expressed as the value of *β* standardized regression coefficient and its significance together with coefficient of determination *R*-squared expressed as the percent of the variation that can be explained by regression equation. A *p* value less than 0.05 was considered statistically significant.

## Results

Table 1 presents the clinical characteristics and biochemical measurements of the 81 participants who completed the study. Maternal clinical characteristics parameters were comparable in both groups except for cigarette smoking habits. In the group of smoking mothers, the mean number of cigarettes per day was 8.8, the mean duration of smoking before conception was 8.6 years, and the mean serum cotinine concentration was 86.5 μg/L. In the tobacco abstinent group, serum cotinine was present only in trace amounts. There were no severe complications during delivery in either group. Mean birth weight, body length, and head circumference of the smoking mothers’ infants were significantly lower (*p* < 0.001; *p* < 0.01; *p* < 0.001, respectively) compared with that of the abstinent group. Apgar score was similar in the two groups. According to biochemical markers, the smoking mothers had significantly lower concentrations of serum hepcidin (*p* < 0.001), iron (*p* < 0.01), and hemoglobin (*p* < 0.05), but higher erythropoietin levels (*p* < 0.05) compared with non-smoking pregnant women. The levels of ferritin, soluble transferrin receptor, and C-reactive protein were similar in both groups.

**Table 1:** Clinical characteristics and biochemical measurements in smoking and non-smoking pregnant women (*n* = 81)

Characteristic	Smoking (*n* = 41)	Non-smoking (*n* = 40)	*p* value
Maternal age (years)^a^	28.8 ± 4.5	30.3 ± 4.8	0.120
Ethnic origin: Caucasian (%)^c^	100	100	–
Maternal weight (kg)^a^	63.9 ± 5.2	65.6 ± 5.4	0.123
Maternal height (cm)^a^	165.1 ± 0.1	165.0 ± 0.1	0.884
Pre-gravid BMI (kg/m^2^)^a^	23.4 ± 1.3	24.1 ± 1.4	0.073
Gestational age of delivery (weeks)^b^	39 [39–40]	39 [39–40]	0.384
Delivery: cesarean/vaginal (%)^c^	4.9/95.1	2.5/97.5	0.571
Neonatal gender female/male (%)^c^	46/54	38/62	0.420
Birth weight (g)^a^			
Whole group	3123.7 ± 426.4	3516.6 ± 445.6	0.000
Male	3160.5 ± 388.7	3647.2 ± 399.2	0.000
Female	3081.1 ± 473.6	3298.7 ± 445.6	0.182
Birth body length (cm)^a^			
Whole group	54.4 ± 2.7	55.9 ± 2.5	0.009
Male	54.6 ± 3.0	56.6 ± 2.4	0.010
Female	54.1 ± 3.0	54.8 ± 2.3	0.431
Head circumference (cm)^a^			
Whole group	34.0 ± 1.7	35.0 ± 1.1	0.000
Male	34.3 ± 1.1	35.2 ± 1.0	0.003
Female	33.7 ± 1.3	34.7 ± 1.1	0.033
Apgar score (5th min)^b^	10 [10–10]	10 [10–10]	0.576
Number of cigarettes/day^b^	10 [5–10]	0	–
Time of smoking before conception (year)^b^	8 [5–12]	0	–
Cotinine (μg/L)^b^	87.9 [58.1–108.5]	0	–
Hepcidin (ng/mL)^a^	10.9 ± 6.8	16.3 ± 6.8	0.000
EPO (mIU/mL)^b^	17.6 [12.5–32.7]	14.4 [12.2–21.3]	0.037
Ft (ng/mL)^b^	11.5 [8.5–15.7]	13.5 [8.8–30.4]	0.170
Fe (μmol/L)^a^	12.0 ± 4.5	16.4 ± 7.0	0.001
Hgb (g/dL)^a^	12.2 ± 1.1	12.7 ± 1.1	0.049
sTfR (μg/mL)^b^	1.3 [1.2–1.7]	1.1 [1.0–1.6]	0.161
Tf (mg/dL)^a^	417.7 ± 65.1	400.1 ± 82.1	0.208
CRP (nmol/L)^a^	59.0 ± 17.1	56.2 ± 16.2	0.549

*BMI* body mass index, *EPO* erythropoietin, *Ft* ferritin, *Fe* iron, *Hgb* hemoglobin, *sTfR* soluble transferrin receptor, *Tf* transferrin, *CRP* C-reactive protein

^a^Values are means ± standard deviation (SD)

^b^Values are median and interquartile range (25th–75th percentiles)

^c^Values are percentage

In smoking pregnant women group, hepcidin was negatively related to the number of cigarettes per day (*p* < 0.01), cotinine (*p* < 0.05), EPO (*p* < 0.05), and sTfR (*p* < 0.05) in linear regression analysis. On the contrary, hepcidin was positively related to Ft (*p* < 0.001) and Hgb (*p* < 0.05). We did not observe significant correlations between hepcidin and iron as well as transferrin. Hepcidin was not related to C-reactive protein—marker of acute phase inflammation (Table 2).

**Table 2:** Univariate regression analyses of hepcidin with number of cigarettes per day, serum cotinine concentration, and studied biochemical markers in smoking pregnant women (*n* = 41)

Independent variables	*β*	SE for β	*p value*	*R* ^2^ %
Number of cigarettes/day	−0.46	0.142	0.002	21.5
Cotinine	−0.38	0.148	0.014	14.5
EPO	−0.25	0.109	0.027	6.1
Ft	0.47	0.099	0.000	22.5
Fe	0.21	0.010	0.054	4.5
Hgb	0.27	0.108	0.015	7.2
sTfR	−0.25	0.109	0.024	6.2
Tf	−0.17	0.111	0.128	2.9
CRP	0.14	0.159	0.399	1.8

*EPO* erythropoietin, *Ft* ferritin, *Fe* iron, *Hgb* hemoglobin, *sTfR* soluble transferrin receptor, *Tf* transferrin, *CRP* C-reactive protein

Correlation studies revealed that birth weight and birth length in infants of smokers were negatively related with serum cotinine and number of cigarettes smoked per day. The relationship between hepcidin concentration and anthropometric parameters was observed in both groups. Additionally, birth body weight was positively correlated with iron and hemoglobin levels only in the group of tobacco abstinent, whereas birth body length correlated negatively with erythropoietin only in the smoking group (Table 3).

**Table 3:** Simple correlations (Spearman or Pearson) between newborn anthropometric parameters and the concentrations of studied biochemical parameters in group of smoking (*n* = 41) and non-smoking (*n* = 40) pregnant women

Biochemical parameters	Birth body weight				Birth body length			
	Smoking group		Non-smoking group		Smoking group		Non-smoking group	
	*r*	*p* value	*r*	*p* value	*r*	*p* value	*r*	*p* value
Hepcidin^a^	0.45	0.003	0.57	0.000	0.47	0.002	0.42	0.008
EPO^b^	−0.21	0.180	−0.10	0.557	−0.42	0.007	−0.06	0.718
sTf ^a^	−0.13	0.427	−0.06	0.708	−0.22	0.175	0.03	0.849
Tf^a^	0.07	0.685	−0.25	0.117	−0.07	0.659	−0.18	0.266
Ft^b^	0.22	0.163	0.30	0.064	0.19	0.233	−0.01	0.967
Fe^a^	0.14	0.393	0.31	0.049	0.20	0.205	0.13	0.420
Hgb^a^	−0.09	0.561	0.32	0.042	0.07	0.669	0.24	0.134
Cotinine^b^	−0.61	0.000	–	–	−0.37	0.017	–	–
Number of cigarettes/day^b^	−0.54	0.000	–	–	−0.32	0.039	–	–

*EPO* erythropoietin, *sTfR* soluble transferrin receptor, *Tf* transferrin, *Ft* Ferritin, *Fe* iron, *Hgb* hemoglobin

^a^Pearson correlation

^b^Spearman correlation

In multi-variable regression model defined for the whole group, adjusted for gender and smoking status, birth body weight was associated with serum hepcidin (*β* = 0.559, *p* = 0.000) and iron (*β* = 0.240, *p* = 0.008) but not hemoglobin (*β* = −0.105, *p* = 0.314). For birth body length, the highest impact of the serum hepcidin concentration was indicated (*β* = 0.496, *p* = 0.000). Markers of estimated intensity of cigarette smoking and ferritin concentration were not included in the same multi-variable model because these parameters and hepcidin were highly dependent (*R*^2^ = 21.5 % for number of cigarettes per day, *R*^2^ = 14.5 % for cotinine, *R*^2^ = 22.5 % for ferritin).

## Discussion

Research on the relationship between smoking during pregnancy and total body iron stores has been conducted for many years, but corresponding literature data are still fragmentary and the results are controversial. No effect of smoking on iron homeostasis parameters during gestation was reported; however, some investigators observed a tendency to decrease concentrations of hemoglobin, hematocrite, and iron with longer smoke exposition and more cigarettes smoked per day [12, 15, 20, 21]. Our findings showed that hemoglobin and iron levels were significantly lower in the smoking group than in the tobacco abstinent. The same results were described by Rasmussen et al. [13] and Zafar et al. [22] who suggested that iron supplementation through pregnancy had a higher relative increase in hemoglobin concentration toward the end of pregnancy. In the case of transferrin, soluble transferrin receptor, and ferritin concentrations, we did not identify differences between the studied groups. This is consistent with other’s previous research where no significant relation between concentration of ferritin and smoking was found [13]. On the contrary, some studies showed higher levels of ferritin in pregnant women who smoke [18, 23]. This may be due to the fact that they have been conducted in group of women between 19 and 26 weeks of gestation, while iron deficiency is the highest at the end of pregnancy [23].

There are a few human studies assessing hepcidin during pregnancy [2, 24–31]. Concentration of this hormone in healthy non-smoking pregnant women decreased gradually in the course of pregnancy in order to maximize iron absorption and availability, thus enhancing transfer to the fetus [4, 26–29]. The third trimester values of hepcidin in our study are consistent with the report by Rehu et al. [30] and Young et al. [26] and slightly higher than the report by other investigators [24, 25, 31]. These differences may result from the method applied with hepcidin, selection of the research group, material, gestational age, and type of delivery. Some authors postulated elevated hepcidin at the day of delivery and in women who delivered vaginally in comparison to cesarean section which may be related to inflammatory cytokine activation during labor and the postpartum period [26, 30, 32]. In general, for uncomplicated pregnancies, no relation between hepcidin level and inflammatory markers during pregnancy was recognized; also our findings are in accordance with these results [1, 25, 31]. Although in both studied group (non-smoking and smoking pregnant women), the C-reactive protein concentrations were slightly higher than in non-pregnant women, it was in the range of gestational age-specific reference values [5]. Similar to Rehu et al. [30] and Young et al. [26], we measured hepcidin in serum of healthy, non-anemic women using ELISA method which directly detects the biologically active 25-amino-acid form of this hormone. It is noteworthy that we conducted our study toward the end of the pregnancy and 90 % of births took place vaginally.

To our knowledge, this is the first study to report influence of tobacco smoking on hepcidin in blood of pregnant women. Previously, we observed a negative effect of smoking on serum pro-hepcidin (pro-hormone) levels during gestation [33]. A recent analysis showed a significant inverse association between hepcidin concentrations and the number of cigarettes per day as well as cotinine levels. Our results confirmed reports observed in groups of non-smoking women regarding the positive association between hepcidin and ferritin as well as hemoglobin during gestation [2, 26–28]. In agreement with Finkenstead et al. [29], also a negative relation between hepcidin and erythropoietin was found. We suggested that accompanying smoking hypoxia can suppress hepcidin mRNA expression reducing this hormone in mothers and probably in newborns. The higher erythropoietin concentration (as indicator of hypoxia) coexisting with lower iron and hemoglobin levels in smoking pregnant women and correlation between this parameter and smoking dose-dependent hepcidin concentration seem to confirm this hypothesis.

As noted in the introduction, hypoxia in smoking people affects not only increased deficiency of iron for erythropoiesis but also oxidative damage depending on the amount of enhanced reactive oxygen species (ROS) generated by tobacco smoke. It is widely accepted that in cigarette smokers, increased oxidative stress is observed, concerning smoking pregnant women [34]. There have been a few in vivo trials documenting the effects of hypoxia on circulating hepcidin level, with only four of those focusing on human. The decreased concentration of this hormone in chronic obstructive pulmonary disease (CODP) patients and in healthy volunteers exposed to hypobaric hypoxia at high altitude was observed [35, 36]. The remaining two suggested that prenatal, utero-placental chronic hypoxia reduced pro-hepcidin synthesis in the fetus [37, 38]. Animal models demonstrate that increasing reactive oxygen species, especially H_2_O_2_, reduced expression of hepcidin mRNA as well as hepatic iron concentration [39, 40]. However, iron, inflammation, and smoking are also known to drive ROS production, so the interaction of these factors is complex and it is likely to be a balance of all this factors that regulates the final level of hepcidin synthesis in human. Therefore, it is possible that oxidative stress may attenuate the iron and inflammation-induced upregulation of hepcidin, which would lead to a relative lowering of hepcidin and elevated iron absorption [11, 39].

There is a strong evidence that smoking during pregnancy influenced negatively on birth body weight, length, and head circumference [41–43]. Also, maternal iron deficiency anemia (IDA) has been linked to higher risk of low birth, but the detailed mechanisms by which this occurs still remain unclear [44–46]. Hepcidin is involved in active transport of iron through placental syncytiothrophoblast from mother to child. The findings of Young and colleagues [26] indicated that maternal hepcidin at least in part determined fetal iron homeostasis. They showed inverse correlation between maternal hepcidin and the iron transport to the fetus [26]. There have been no studies that describe association between maternal concentrations of hepcidin and birth anthropometric parameters. Some authors reported that cord blood hepcidin level is similar in full-term intrauterine growth-restricted and appropriate-for-gestational-age infants at birth [9]. However, maternal hepcidin concentration was not associated with cord blood hepcidin in a newborn at term [30]. We found that maternal hepcidin could be an important predictor of birth weight and length of full-term newborns in tobacco abstinent as well as in smoking. Maternal smoking is associated with increased fetal iron requirements and stimulates fetal erythropoiesis depending on the degree of exposure to tobacco smoke [16, 46, 47]. Maternal low level of hepcidin and negative iron balance may lead to intrauterine growth retardation as well as low birth weight [9, 30, 45]. Further studies may explain the mechanism by which downregulation of hepcidin by tobacco smoking hypoxia contributes to fetal growth.

Our study is limited by its relatively small sample size; however, both studied groups were similar for ethnicity, gestational age, fetal gender, and type of birth which are recognized as important factors for hepcidin levels [1, 30, 32, 48]. Additionally, we did not measure iron dietary intake, which might influence markers of iron metabolism, but our population consisted of healthy, well-nourished mothers remaining on a mixed diet and receiving regular multivitamin preparation (containing small doses of iron and folic acid) as a part of routine perinatal care. Another limitation is that we have not studied direct effect of tobacco smoking on newborn iron metabolism, and future research should assess maternal and cord blood hepcidin level in a large group of matched maternal cord pairs.

In conclusion, tobacco smoking affected hepcidin level in serum of pregnant women in a dose-dependent manner. Low concentrations of iron and hemoglobin in maternal serum coexisting with high level of erythropoietin suggest that smoking could lead to subclinical iron deficiency and chronic hypoxia not only in mothers but also in fetus. Low serum hepcidin concentration in smoking pregnant women might be associated with lower fetal birth weight and length.
